# Identification of regulatory modules in genome scale transcription regulatory networks

**DOI:** 10.1186/s12918-017-0493-2

**Published:** 2017-12-15

**Authors:** Qi Song, Ruth Grene, Lenwood S. Heath, Song Li

**Affiliations:** 1 program in Genetics, Bioinformatics and Computational Biology, Virginia Polytechnic Institute and State University, Blacksburg, VA 24061 USA; 2 Department of Plant Pathology, Physiology, and Weed Science, Virginia Polytechnic Institute and State University, Blacksburg, VA 24061 USA; 3 Department of Computer Science, Virginia Polytechnic Institute and State University, Blacksburg, VA 24061 USA; 4 Department of Crop & Soil Environmental Sciences, Virginia Polytechnic Institute and State University, Blacksburg, VA 24061 USA

**Keywords:** Co-regulation, Network module, Regulatory network, Systems biology

## Abstract

**Background:**

In gene regulatory networks, transcription factors often function as co-regulators to synergistically induce or inhibit expression of their target genes. However, most existing module-finding algorithms can only identify densely connected genes but not co-regulators in regulatory networks.

**Methods:**

We have developed a new computational method, CoReg, to identify transcription co-regulators in large-scale regulatory networks. CoReg calculates gene similarities based on number of common neighbors of any two genes. Using simulated and real networks, we compared the performance of different similarity indices and existing module-finding algorithms and we found CoReg outperforms other published methods in identifying co-regulatory genes. We applied CoReg to a large-scale network of *Arabidopsis* with more than 2.8 million edges and we analyzed more than 2,300 published gene expression profiles to charaterize co-expression patterns of gene moduled identified by CoReg.

**Results:**

We identified three types of modules in the *Arabidopsis* network: regulator modules, target modules and intermediate modules. Regulator modules include genes with more than 90% edges as out-going edges; Target modules include genes with more than 90% edges as incoming edges. Other modules are classified as intermediate modules. We found that genes in target modules tend to be highly co-expressed under abiotic stress conditions, suggesting this network struture is robust against perturbation.

**Conclusions:**

Our analysis shows that the CoReg is an accurate method in identifying co-regulatory genes in large-scale networks. We provide CoReg as an R package, which can be applied in finding co-regulators in any organisms with genome-scale regulatory network data.

**Electronic supplementary material:**

The online version of this article (10.1186/s12918-017-0493-2) contains supplementary material, which is available to authorized users.

## Background

**Fig. 1:**
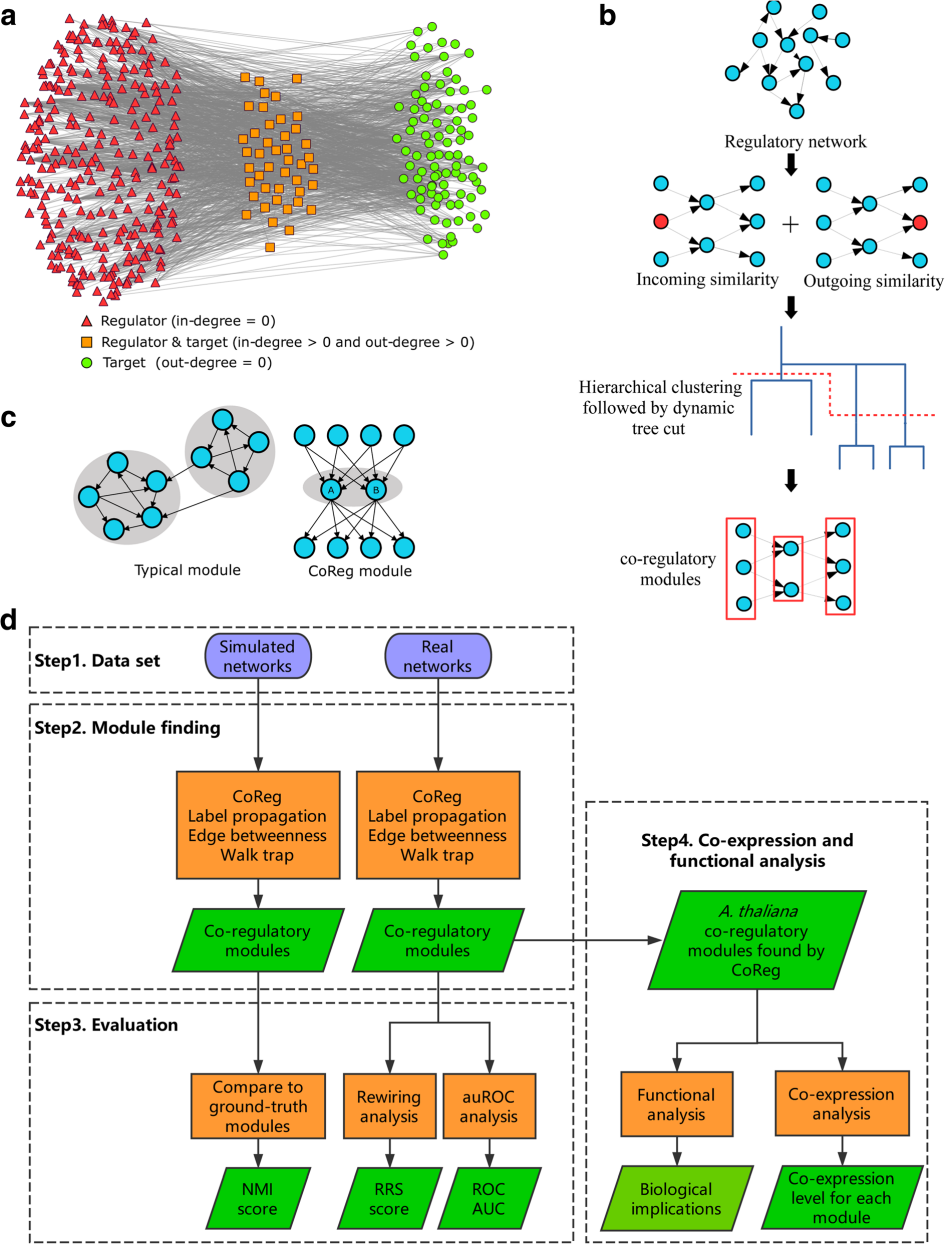
The complexity of the *A. thaliana* regulatory network, two clustering strategies and the work flow of CoReg. **a** The complexity of regulatory *A. thaliana* network. Each node represents one gene in the network and each edge represents an interaction between one TF and its target. We classified the nodes into three categories based on the degree: 1) triangle, in-degree = 0; 2) rectangle, in-degree >0 and out-degree >0; 3) circle, out-degree = 0. **c** CoReg uses a clustering strategy different from existing clustering method. Typically, the network modules that normal clustering algorithm identifies are shown on the left. However, if there are two genes which share many targets and regulators in common, they are most likely to be the actual co-regulators (shown on the right, gene A and gene B) CoReg is designed to work on the clustering problem on the right. **b** The brief work flow of CoReg starting from input (a regulatory network). Red nodes in the second step represent common target (for out-similarity) or regulator (for in-similarity) for the pair of nodes in the middle. CoReg adds up the incoming similarity and outgoing similarity and then calculates a distance matrix. Next, distance matrix is used as the input to hierarchical clustering. In the last step, dynamic tree cut is performed to obtain final module assignment for each node. **d** flowchart of CoReg analysis

Characterization of the structures of gene regulatory networks is an essential step towards understanding transcriptional regulation in living organisms. In recent years, genome scale regulatory networks have become available for many species [1–6]. In the human ENCODE project, transcription factor (TF)-target interactions for 119 human TFs have been identified using Chromatin Immunoprecipitation followed by sequencing (ChIP-seq) [1]. In the model plant species *Arabidopsis thaliana*, cell type-specific regulatory networks in xylem and ground tissues were generated using enhanced yeast one-hybrid (eY1H) for 267 TFs [4, 5]. Genome-scale TF-target interactions can also be inferred from direct sequencing of TF binding sites in vitro [7] and by measuring TF binding specificity [8]. Recently, ePlant platform has provided the integration of interaction data and convenient access to TF-target interaction data for plant research [9]. Co-expression and TF-binding site based prediction is another approach to infer TF-target interactions, which has been successfully implemented in TF2Network [10]. These experimentally identified or predicted gene regulatory networks typically contain thousands of nodes and thousands to millions of edges (Fig. 1a) [1, 7], which provide much information regarding the regulatory targets of each TF and the putative regulators of each gene in the genome. A key challenge is how to use these large-scale networks to identify functional information for both TFs and their target genes.

One way to approach this problem is to find clusters of genes with similar regulatory properties [11–13]. In *Arabidopsis*, it has been shown that identification of co-regulatory modules can provide insight into biological function [5]. For example, analysis of a regulatory network showed that two key transcription factors (SHORTROOT and SCARECROW) that determine cell fates in ground tissues are controlled by both activators and repressors [5]. Co-regulatory targets of stress-responsive transcription factors were found to be key regulators of ABA responses [14]. In general, a regulatory network is represented by a directed graph and the process of identifying clusters of nodes (regulatory modules) with similar network properties is called network module finding [15] or modular decomposition [16]. Many computational approaches have been developed for module finding, and these approaches are based on various ways to calculate node similarities followed by graph partitioning methods. For example, Walk Trap (WT) [17] calculates the distance between nodes and groups nodes based on pairwise similarity matrices; Edge Betweenness (EB) [18] builds hierarchical relationship between the nodes and partitions the network into modules; Label Propagation (LP) [19] performs simulation on the network by propagating cluster labels. Other examples include leading eigenvectors [20] and spin-glass [18]. These algorithms can be applied to either undirected networks [17–20] or directed networks [15] and, in many cases, performed well in finding groups of densely connected nodes. However, densely connected groups may not reflect biologically meaningful clusters in regulatory networks. For example, in Fig. 1c, gene A and gene B are biologically related because they regulate the same target genes and will be expected to form one network module, which is not the typical module that most clustering approaches are designed to identify (Fig. 1c).

Here, we propose a new computational tool, CoReg, to identify co-regulatory modules in genome scale regulatory networks. CoReg calculates the similarity of genes based on their common targets and regulators and groups highly similar genes into co-regulatory modules. We compared several similarity indices, including the Jaccard index, the geometric index, and the inverse log-weighted similarity index using simulated and real networks (Fig. 1b and d). In simulated networks, we tested CoReg with different module sizes. We also performed extensive rewiring-simulation and tested CoReg on plant, human, and bacterial networks. CoReg outperformed other commonly used module finding methods in identifying co-regulatory modules in all data sets tested. We identified many co-regulatory modules in the *Arabidopsis* genome and demonstrated that the expression levels of genes in some of the modules are also highly correlated. Finally, we applied CoReg to published gene expression data in *Arabidopsis* and found that gene co-regulatory modules tend to be highly co-expressed in abiotic stress conditions. CoReg is implemented as an R package, which can be used to analyze any regulatory network. Sample network data used in this paper and the CoReg package can be downloaded from GitHub (https://lilabatvt.github.io/CoReg/).

## Results

### Assessment of different module finding methods

There are typically two approaches to evaluate a computational method: using either existing biological knowledge or using computational simulations as a “gold standard”. Since there has not been a systematic study that summarizes known co-regulatory modules in any species, we performed computational evaluations in two ways: 1) we generated simulated networks with pre-specified module assignment for each node and evaluated different methods using mutual information; and 2) we performed duplication-rewiring simulation on real networks and evaluated the results using receiver operating characteristic (ROC) curves and rewiring recall score.

### Performance assessment using simulated networks

We generated the simulated networks using a method described in a previous publication [21]. We modified this approach to generate co-regulatory modules for directed networks (See Methods). Simulated networks were generated using different combination of parameters to explore the performance of algorithms in varying module size and number of targets (see Methods for details). Briefly, each regulator node was assigned to predefined modules. A pool of candidate targets was selected for each module. Each regulator node can link to a node either in the pool or a node outside the pool according to a fixed probability. This procedure was repeated until target nodes for each module had been assigned. One of the key parameters is the probability *prob*. With higher *prob*, the generated modules will have a stronger co-regulation pattern, characterized by nodes in co-regulatory modules connecting to a small group of nodes rather than random targets in the network (Additional file 1: Figure S1). We then tested different module-finding algorithms on the simulated network. The performance was evaluated by comparing the algorithm identified modules to the pre-specified modules using the Normalized Mutual Information (NMI) score [22].

**Fig. 2:**
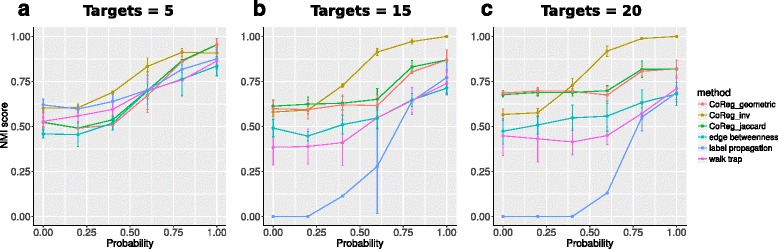
Evaluation of different module-finding methods using simulated networks. Each data point is the average score of five runs. We constructed simulated networks with module size of 5. Simulation result of other tested parameters are in Additional file 2: Figure S2. **a** Number of targets is equal to 5. **b** Number of targets is equal to 15. **c** Number of targets is equal to 20

The NMI score (see Methods) between the pre-specified modules and algorithm identified modules was plotted against the co-regulation probabilities. We plotted the NMI score curve for each similarity index used by CoReg: 1) CoReg with inverse log weighted similarity index, (CoReg + inv.); 2) CoReg with jaccard index (CoReg + jaccard); and 3) CoReg with geometry similarity index (CoReg + geometric). The three methods were compared to the result generated by Walk Trap (WT), Label Propagation (LP), and Edge Betweenness (EB). Figure 2 shows the NMI scores under different parameters. In all the simulations, the NMI scores show an increasing trend as the co-regulation probability increases, indicating that algorithms perform better when the network has a stronger co-regulation pattern. This trend is apparent for all the methods tested using simulated networks except for CoReg + inv. The NMI scores for CoReg + inv. decrease when co-regulation probability is greater than 0.6 (Additional file 2: Figure S2), which is due to the high similarity between target genes (see Discussion). These results showed that CoReg + jaccard and CoReg + geometric consistently outperformed the other methods (Fig. 2).

### Performance assessment using real networks

For real networks, we designed our simulation such that the simulated networks are based on the known topology of biological networks. We used published regulatory networks from human, *Arabidopsis,* and *Escherichia coli* (*E. coli*) as the starting point for our simulations (see Methods). In each simulation, we selected a subset of regulators and duplicated those genes, while preserving their neighbors in the network. We then rewired the network with a pre-specified probability to introduce noise to the network (for more details, see Methods and Additional file 11: Figure S5). For each species, we tested three rewiring probabilities (0.1, 0.3 and 0.5). In the simulated networks, a gene and its duplicated counterpart belong to the same co-regulatory module, and these genes are used to evaluate algorithm performance using receiver operating characteristic (ROC) and area under the ROC curves (auROC).

**Fig. 3:**
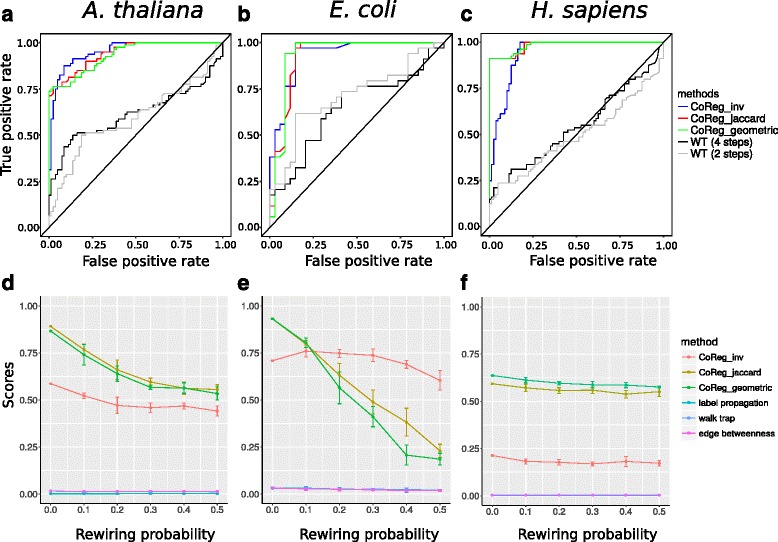
Evaluation of the different module-finding methods using real networks. We used different similarity indices for CoReg, CoReg_inv: CoReg + inverse log weighted similarity index; CoReg_jaccard: CoReg + jaccard similarity index; CoReg_geometric: CoReg + geometry index. CoReg was also compared to other three clustering algorithms, namely, Label Propagation (LP), Edge Betweenness (EB), Walk Trap (WT). We performed the evaluation on *A. thalina*, *E. coli* and *H. sapiens* network, respectively (From left to right, species was indicated on the top of the figure). **a, b, c** The ROC curve for co-regulators pairs based on the ranking result from CoReg and WT. **d, e, f** Rewiring recall score for all the methods. We calculated rewiring recall score under rewiring probability from 0 to 0.5. Each data point is the average score of five runs. Error bar was added to show the standard error. For the human network*,* EB algorithm was not tested because computation cannot be finished within a reasonable amount of time on large-scale network such as human network

For each species, we plotted the ROC curves for CoReg + inv., CoReg + jaccard, and CoReg + geometric. These similarity indices were compared to the similarity index computed from Walk Trap (**WT**). **LP** and **EB** are not used here because these two methods do not calculate a similarity matrix and cannot be directly compared. WT allows the user to specify the length of random walks. We tested WT with steps = 2 and steps = 4. Figure 3a, b, and c show the ROC curves generated from the three species with rewiring probability = 0.5, where CoReg outperforms WT in all simulations. ROC curves for the three similarity indices have very similar performance. The AUC values of CoReg + jaccard and CoReg + geometric are always slightly higher than those of CoReg + inv. (Table 1).

**Table 1:** AUC for CoReg with different similarity index and Walk Trap. Highest values in each row is in bold

Species	Rewiring probability	CoReg inv	CoReg jaccard	CoReg geometric	WT (4 steps)	WT (2 steps)
*A. thaliana*	0.1	0.859 ± 0.035	0.957 ± 0.017	**0.972 ± 0.014**	0.597 ± 0.049	0.556 ± 0.033
*A. thaliana*	0.3	0.823 ± 0.031	**0.939 ± 0.010**	0.910 ± 0.036	0.548 ± 0.048	0.526 ± 0.027
*A. thaliana*	0.5	0.794 ± 0.035	**0.872 ± 0.034**	0.866 ± 0.031	0.531 ± 0.027	0.505 ± 0.038
*E. coli*	0.1	0.849 ± 0.048	0.968 ± 0.022	0.963 ± 0.013	0.611 ± 0.030	**0.971 ± 0.020**
*E. coli*	0.3	0.813 ± 0.037	**0.930 ± 0.023**	0.919 ± 0.027	0.557 ± 0.035	0.827 ± 0.034
*E. coli*	0.5	0.819 ± 0.033	**0.865 ± 0.024**	**0.878 ± 0.039**	0.555 ± 0.028	0.683 ± 0.060
*H. sapiens*	0.1	0.914 ± 0.037	**0.999 ± 0.001**	**0.999 ± 0.002**	0.500 ± 0.033	0.512 ± 0.025
*H. sapiens*	0.3	**0.919 ± 0.035**	0.994 ± 0.007	0.991 ± 0.008	0.516 ± 0.016	0.489 ± 0.025
*H. sapiens*	0.5	0.918 ± 0.032	**0.983 ± 0.010**	**0.983 ± 0.008**	0.512 ± 0.027	0.503 ± 0.025

CoReg inv.: CoReg + inverse log weighted similarity index

CoReg jaccard: CoReg + jaccard similarity index

CoReg geometric: CoReg + geometry similarity index

WT: Walk Trap

### Rewiring recall score

The second step in finding co-regulatory modules is node clustering. To assess and compare the performance of different clustering methods, we calculated rewiring recall scores (**RRS**) for all clustering methods and compared the results obtained using different methods. The rewiring recall score is a normalized measure of the accuracy of the method. For an ideal clustering method, each duplicated node and its original node should belong to the same module with only these two nodes in this module. The RRS is designed to equal 1 under such an ideal cluster assignment (see Methods). If a method can find a module containing both the duplicated node and its original node, but the module also includes other nodes, the score will be less than 1 (see Methods). In fact, RRS can be very small if the module includes large number of nodes (Additional file 3: Figure S3). In our simulations, RRS rarely equals 1, because if two genes are regulating the same set of targets in the original network, the duplicated simulation will introduce another gene that is highly similar to both genes. In this situation, the RRS cannot equal 1 for the correct clustering. Despite this limitation, RRS can be used to compare relative performance between different methods.

In the *A. thaliana* network, the CoReg + geometric index and CoReg + jaccard index outperformed all other clustering methods, and their performances are similar to each other (Fig. 3d). In the *E. coli* network, both the CoReg + geometric index and the CoReg + jaccard index have better performance than other methods when rewiring probability is equal to 0 and 0.1. However, as the rewiring probability increases, performance of CoReg + jaccard and CoReg + geometric drops much faster than that of CoReg + inv. (Figure 3e), and CoReg + inv. started to outperform CoReg + jaccard and CoReg + geometric when rewiring probability equals 0.2. The RRS scores for other methods (WT, LP, and EB) also slightly decrease as rewiring probability increases (see Additional file 3: Figure S3). In the *H. sapiens* network, the decreasing trend of performance is not very obvious as compared to the other two species, presumably due to the large size of the human network. Although no single similarity index performed better than all others in all species, in our simulations, CoReg + jaccard and CoReg + geometric outperformed CoReg + inv. more often than CoReg + inv. outperformed CoReg + jaccard and CoReg + geometric. In the *A. thaliana* and *E. coli* networks, CoReg + jac outperformed CoReg + geo.

### Different tree cut strategies: Dynamic tree cut and static tree cut

**Fig. 4:**
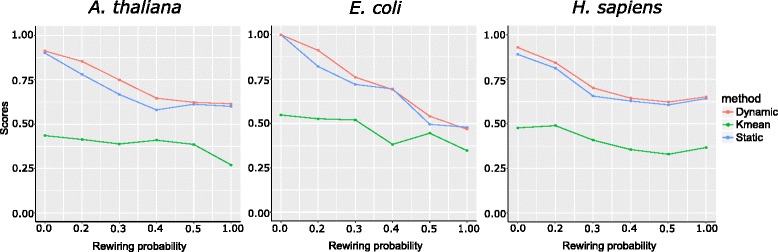
Comparison of dynamic tree cut and static tree cut. Different tree cut method was applied after hierarchical clustering. We evaluated each tree cut method by RRS

A proper strategy to cut the hierarchical tree is necessary because 1) there is no prior knowledge available for the expected number of modules and 2) it is really difficult to decide an optimal cutting height that works for all the branches of the hierarchical tree. The parameters provided by the dynamic tree cut algorithm gives more parameters to adjust module size (see Methods), providing flexibility to tree cutting. Here, we explored the performance of both static tree cut and dynamic tree cut strategies for cutting a hierarchical tree. The performance of each method was shown in Fig. 4. For all three species, dynamic tree cut has outperformed the static tree cut in most of the rewiring probabilities. Thus, in the case of co-regulatory modules finding, dynamic tree cut performs better than static tree cut.

### CoReg identified three types of co-regulatory modules

After computational simulation and comparisons, we decided to use CoReg + jaccard in the following analysis because of the consistent performance of this similarity measurement. CoReg identified 87, 141, and 1208 co-regulatory modules in *A. thaliana*, *E. coli,* and *H. sapiens* networks, respectively. We focused on the *A. thaliana* network for further explorative analysis, because we are interested in the roles of co-regulatory genes in plant development and abiotic stress responses and in regulatory connections between those two processes. For the *A. thaliana* network, the largest co-regulatory module contains 13 nodes, while the smallest module contains only two nodes. To annotate the transcription factors in this network, we obtained transcription factor annotations from the Plant Transcription Factor Database (PlantTFDB) [23]. The co-regulatory module assignment and protein family assignment for each transcription factor are provided as Additional file 4: Table S1 For each co-regulatory module, we identified all the genes within the module and their first neighbors in the network. All the interactions between these genes and gene annotations are presented in Additional file 5: Table S2.

**Fig. 5:**
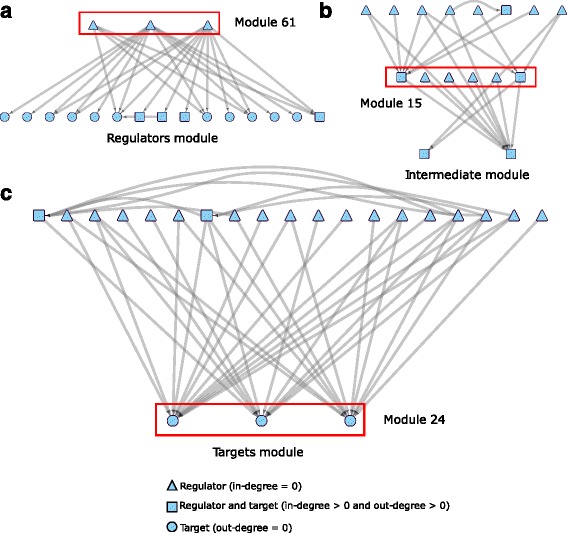
Three types of modules identified by CoReg. Based on the in-degree and out-degree of the genes in each module, modules were classified into three categories. Red boxes indicate nodes in the same module. In each of the panels, only first neighbors of the nodes in the modules are included. **a** Regulator modules. **b** Intermediate modules. **c** Target modules. Please see Additional file 5: Table S2 for the gene names for each module

Based on the in-degree and out-degree of the genes in the co-regulatory modules, co-regulatory modules can be classified into three types: 1) regulator modules, which include genes with more than 90% of the edges as out-going edges; 2) target modules, which include genes with more than 90% of the edges as incoming edges; and 3) other modules, which are classified as intermediate modules. A regulator module mostly consists of regulators, which are likely to initiate transcriptional regulation, whereas a target module contains mostly target genes of transcriptional regulation. An intermediate module serves as a mediator for regulation activities. Figure 5 shows examples for each type of module. The regulator module in Fig. 5a contains three regulators from module 61 (AT2G38340, AT5G15210, and AT1G24625). The intermediate module in Fig. 5b consists of 6 genes from module 15 (AT5G44080, AT3G49930, AT2G31370, AT1G32150, AT1G09540 and AT2G22850), which connect to 8 regulators and 2 targets. The three regulators connect to 16 target genes in total. The target module shown in Fig. 5c includes three target genes from module 24 (AT5G17420, AT5G13180, and AT5G44030), which are targeted by 19 TFs in total. Additional file 5: Table S2 shows the module ID for each gene. The presence of many common targets and common regulators demonstrates the co-regulation detected by CoReg in complex directed networks.

### CoReg identified both known and novel co-regulatory modules

Because the co-regulatory modules are solely based on their network connections, we investigated the expression patterns of genes in the same co-regulatory modules using published microarray expression data from the AtGenExpress database [24–26]. The expression of over 22,000 *Arabidopsis* genes was analyzed using microarray hybridization and provided expression patterns in three data sets: a developmental tissue series, hormone treatments, and abiotic stress responses. In the case of the developmental tissue series, various tissue types including leaves, roots, flowers, and stems were sampled at different developmental stages. In the case of the hormone treatment data set, plant hormones--auxin, cytokinin, gibberellin, brassinosteroid, abscisic acid, jasmonate, and ethylene--  were used to treat growing seedlings, and expression levels were monitored in time course experiments. In the abiotic stress data set, time course experiments were performed under abiotic stress conditions, including heat, cold, drought, salt, high osmolarity, UV-B light, and wounding (see Methods). To measure the correlation between the genes in co-regulatory modules in the *A. thaliana* network, we calculated the Pearson Correlation Coefficient (PCC) between genes and estimated the significance of PCC for each co-regulatory module using these three different data sets. Twenty-one out of 87 modules identified by CoReg show significant co-expression (estimated *P*-value <0.05) in at least one of the three data sets. More specifically, there are 6, 13, and 4 modules showing significant co-expression in the developmental, stress, and hormone expression data sets, respectively. These results suggest that genes in co-regulatory modules are co-expressed across various conditions and play roles in transcriptional co-regulation.

**Fig. 6:**
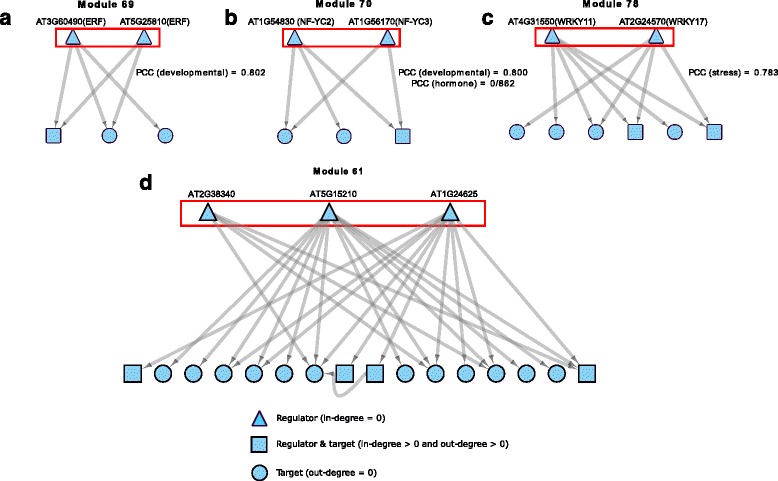
Visualization of module 69,70 and 78, along with their first neighbors in the network. **a** Module 69 and all of its first neighbors. **b** Module 70 and all of its first neighbors. **c** Module 78 and all of its first neighbors. **d** Module 61 and all of its first neighbors

Among all modules identified by CoReg, module 69 includes two ethylene response factor (ERF) transcription factors: AT3G60490 and AT5G25810 (Fig. 6a). Genes in the ERF family play important roles in various developmental and physiological processes in plants [27], such as leaf petiole development [28], shoot formation [29], resistance to pathogen attack [30], and various abiotic stresses [31]. Co-regulation between AT3G60490 and AT5G25810 was not previously reported. AT3G60490 and AT5G25810 show a significantly high correlation with each other in the developmental data set (PCC = 0.802, *P*-value <0.05), suggesting that module 69 is a co-regulatory module involved in developmental processes.

CoReg also identified module 78, which contains two WRKY transcription factors (Fig. 6c). The members of the WRKY transcription factor family are involved in diverse biological processes, such as response to biotic/abiotic stresses, seed development, and seed germination [32]. The two transcription factors, AT4G31550 (WRKY11) and AT2G24570 (WRKY17), are previously reported to be involved in the regulation of basal defense against *Pseudomonas syringae* pv tomato [33]. It was concluded that both WRKY11 and WRKY17 act as negative regulators in this defense process, and WRKY11 and WRKY17 double mutant plants showed stronger defense than WRKY11 single mutant plants [33]. Expression analysis shows that this co-regulatory module has a high expression correlation (average PCC = 0.783, *P*-value <0.05,) in the stress data set, suggesting both WRKY11 and WRKY17 are active during abiotic stress response process. The results from module 70 and 78 indicate that our method can identify known co-regulatory modules through mining large-scale gene regulatory networks. We therefore analyzed other CoReg modules to identify significantly co-expressed modules.

Among other modules identified by CoReg, module 70 (Fig. 6b) contains two transcription factors from the nuclear factor YC (NF-YC) gene family: AT1G54830 (NF-YC3) and AT1G56170 (NF-YC2). NF-Y is a transcription factor complex that includes subunit A, B and C. The three subunits form a NF-Y transcription factor complex that binds to promoters containing a CCAAT-box [34, 35]. NF-YC2 and NF-YC3 were found to participate in the control of floral induction in *A. thaliana* [35]. Our expression analysis shows that the two NF-YC TFs are significantly highly correlated with each other in the developmental data set (PCC = 0.800, *P*-value <0.05) and in the stress data set (PCC = 0.862, *P*-value <0.05). The developmental data set covers a broad range of developmental stages from embryogenesis to senescence, which suggests that co-regulation of NF-YC2 and NF-YC3 may also participate in stress response and other developmental processes.

Module 61 contains three TFs (Fig. 6d). Two of them are AT5G15210, encoding DREB19, which is active both in development and in stress responses [36], and AT2G38340, encoding ZFHD3/HB30, a homeodomain protein with a role in the regulation of floral development [37]. DREB19 had 12 targets in the module, while ZFHD3 had only five targets, four of which were co-regulated by DREB19. The four co-regulated targets are each associated with growth and development: BLH3, a TF that regulates the transition from vegetative to reproductive development [38]; PGSIP1, a protein involved in secondary wall biosynthesis; AT4G28370, encoding an E3 ligase associated with plant cell wall modification; and AT2G34710, encoding an HD Zip TF, PHB, which regulates leaf vascular development through auxin responses.

### CoReg reveals roles of co-regulatory modules in *Arabidopsis* abiotic stress responses

**Fig. 7:**
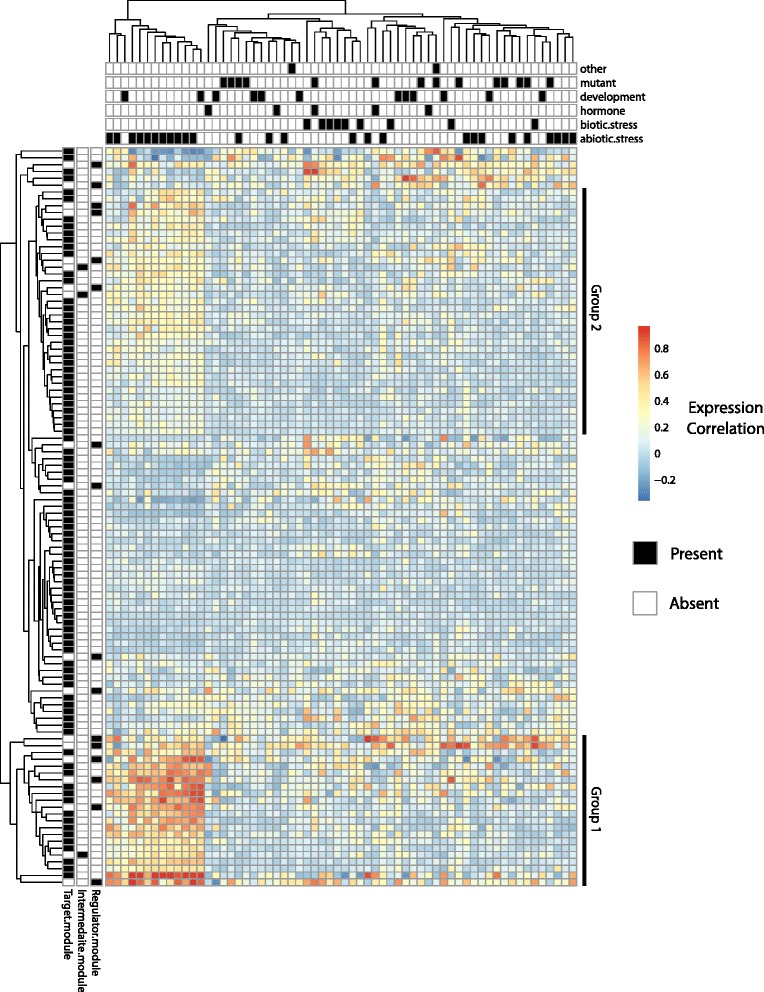
Heat map for co-expression levels of CoReg-identified modules in DAP-Seq network. We first ran CoReg on the DAP-seq network to identify the modules. Then for each co-regulatory module, we calculated the pairwise co-expression values for all the genes in the module and averaged the pairwise co-expression to get a single co-expression value for the module. We selected 108 modules which are significantly co-expressed (*p*-value <0.05) in at least 20 out of 62 experiments (See estimate *p*-value in large-scale network in Methods section) to plot the heat map for co-expression. The conditions for each experiment are marked in the top panel with black squares. The module type is marked on the left panel with black square

To test whether genes in CoReg modules are also co-expressed in a genome-scale network, we applied CoReg to a large scale regulatory network generated by DAP-seq with more than 2.8 million interactions and more than 2300 gene expression profiles (Fig. 7). We applied CoReg to the DAP-seq network to identify co-regulatory modules. Then, for each co-regulatory module, we calculated the pairwise PCCs for all the genes in the module and obtained a module average PCC.

The gene expression data (2327 samples) were generated from 62 experiments with each experiment containing multiple replicated samples. These 62 experiments fell into multiple categories, including biotic stresses, abiotic stresses, hormone treatments, developmental series, and mutant experiments. One experiment can be assigned to multiple categories. Experiments that do not fall into the categories listed above are classified as “other types”. Information on experimental conditions for each experimental data set is shown in Additional file 6: Table S3, and each experiment has a unique GSE id from the gene expression omnibus (GEO) database. We selected the 108 modules whose co-expression is significant (*p*-value <0.05) in at least 1/3 of all 62 experiments and plotted the PCC values for these 108 modules across different treatments (Fig. 7). The co-expression values for all 108 modules in different GSE accessions and the corresponding *p*-value for these co-expression values are provided as Additional file 7: Table S4 and Additional file 8: Table S5, respectively. Among these modules, we found two major groups of coReg modules. Group 1 contains 15 target modules, 1 intermediate module, and 6 regulator modules. Group 2 contains 30 target modules, 2 intermediate modules, and 4 regulator modules. In both groups, genes within the modules are highly co-expressed in abiotic stress conditions, suggesting that modules identified by coReg are likely to be co-expressed under abiotic stresses in *Arabidopsis*.

## Discussion

Two recently published online tools, TF2Network [10] and ePlant [9], have integrated DAP-seq data and TF binding sites for regulatory network prediction and visualization. TF2Network is an online tool, which allows the user to infer candidate regulators from a list of genes. ePlant provides a user-friendly interface for query and visualization of regulations predicted by DAP-seq experiments. To compare the results produced by CoReg to TF2Network and ePlant, we selected all target genes of module 61 to infer their regulators in TF2Network and ePlant. TF2Network identified 49 regulators for the given list of targets, while CoReg identified 3 co-regulators for the same set of targets. We did not find any overlap between TF2Network identified regulators and CoReg identified co-regulators. This difference is likely because TF2Network method used TF binding sites to infer interactions whereas CoReg used direct interactions. However, CoReg can be applied to any network such as a network predicted by the TF2Network method. For ePlant, we submitted three co-regulators in module 61 to search for their targets. ePlant identified 9620 targets in total, while there are 17 targets for module 61 in our network. There are 11 targets that can be found by both ePlant and CoReg. This result shows that, while ePlant provides an overview of all potential targets of given TFs, CoReg selects the subset of targets that are commonly regulated by these TFs.

The three similarity indices calculated the similarity score by measuring the proportion of overlap of first neighbors. The difference is that inverse log-weighted index takes into account the degrees of shared first neighbors while the other two do not. The idea is that two nodes may be more similar if they share some common low-degree neighbors, because high-degree neighbors are more likely to connect to the nodes by pure chance [39]. However, from our simulation result, this strategy led to decreased performance when co-regulation probability is high (*prob > 0.6,* Additional file 2: Figure S2), and when the module size is larger than or equal to the number of targets (e.g. module size = 15 or 20 and target = 5). In these cases, regulators in the same module share few targets, whereas targets have higher degrees than the regulators. The inverse log-weighted similarities between targets are thus higher than the similarities between regulators, which causes CoReg to fail to identify co-regulators in the same module. In contrast, the Jaccard index and the geometric index are normalized by the total number of common neighbors and the product of number of common neighbors, respectively. These methods avoid the problem in the inverse log-weighted similarity. Our results suggest that the CoReg + geometric and the CoReg + jaccard are better choices when the number of regulators is larger than the number of targets. However, such situations are unlikely to happen in transcription networks because the number of transcription factors is usually much smaller than the number of target genes.

Combinatorial regulation by transcription factors (TFs) of target genes underlies the functioning of gene regulatory networks and determines gene expression levels during development and under both biotic and abiotic stresses in many organisms. In *Arabidopsis* and rice, WRKY transcription factors are found to form four and nine co-regulatory clusters respectively [40]. These clusters are involved in diverse signal transduction pathways and in pathogen responses [40]. In a prokaryotic organism such as *E. coli*, transcriptional co-regulation is a key mechanism, for example, in regulating cellular responses to changes in amino acid pools [41]. In human studies, co-regulation was found to be significantly enriched in gene regulatory networks and to be important for maintaining the robustness of gene regulation [42]. Co-regulation is also involved in specific disorders in human; for example, mis-regulation of two co-regulators was shown to be related to the onset of autism [43]. Transcriptional co-regulators are also mis-regulated in breast and ovarian cancer [44]. These studies show that co-regulators occur ubiquitously in all living organisms and are involved in many biological processes, and our computational method represents one important step towards identifying all tissue- and condition-specific co-regulatory modules.

In recent years, high throughput experimental techniques, such as yeast one hybrid [4, 5], ChIP-seq [1, 14] protein binding microarray (PBM) [8], and DNA affinity purification sequencing (DAP-seq) [7], have significantly increased the number of known transcriptional regulatory networks for many organisms. We have tested CoReg in three different organisms and on networks generated by four technologies, including ChIP-seq, DAP-seq, Yeast-1-hybrid, and a literature database. Our results showed that CoReg performed better than existing approaches for all these species and methods. These powerful experimental techniques will provide CoReg with abundant data sets to mine co-regulation information in the future.

Besides direct TF-gene interactions analyzed in this study, other genomic data, such as chromatin modification data and DNA methylation data, are also available for many species. Both chromatin modifications and DNA methylations are known to regulate gene expression. However, the challenge is that both chromatin modification and DNA methylation data are condition- and cell type-specific. Therefore, we did not include chromatin modification or DNA methylation in our analysis. Multiple other methods exist for the incorporation of other types of regulatory information in CoReg. For a pair of regulatory nodes in the network, the information of chromatin modification or DNA methylation of the target genes can be directly added to the similarity measurement used by CoReg. Alternatively, other regulatory information can be analyzed in a post-hoc fashion: for co-regulatory modules identified by CoReg, one can perform enrichment analysis to identify which type of chromatin modification or DNA methylations are enriched in the co-regulatory modules.

Using *Arabidopsis* as a model system, we found that CoReg not only detected known co-regulatory genes such as WRKY transcription factors but also uncovered previously unknown co-regulatory genes. By integrating gene expression data with regulatory network information, we identified co-regulatory modules that are highly co-expressed under abiotic stresses and hormone treatments and during plant development. These results suggest that CoReg can be used to mine existing network data and gene expression data to identify key co-regulatory genes in many other organisms.

We identified three types of co-regulatory modules: regulator modules, target modules, and intermediate modules. By combining CoReg modules with gene expression data from almost all published studies in *Arabidopsis* [45], we found that target modules tend to be highly co-expressed under abiotic stress conditions. For example, in module 24 (Fig. 5c), there are three target genes co-regulated by 19 regulators, with each of the three genes regulated by more than 10 regulators. This observation could represent one type of structural stability in gene regulatory networks where the network structure is robust against perturbations because removal of each individual edge or regulatory nodes has only a small impact on the total number of regulators for each target gene. In contrast, in regulator modules (for example, module 61 in Fig. 5a), each regulator is regulating many genes and mutations in any of the regulators can have a strong effect on expression of the target genes. Genes in module 24 and module 61 are highly connected hub genes. However, in directed gene regulatory networks, perturbation of regulators of target modules has a small impact on expression regulation. This may explain why target modules are widely used in abiotic stress responses in *Arabidopsis*.

## Conclusion

In this study, we developed a computational tool, CoReg, for identifying co-regulators in gene regulatory network. We performed simulation-based analysis to evaluate CoReg and other module-finding algorithms. The results show that CoReg outperforms other algorithms in identifying co-regulators. We applied CoReg to a genome-scale regulatory network for *Arabidopsis*. Results obtained from the subsequent co-expression analysis using a large expression data set indicates that many highly co-expressed modules in this network are associated with abiotic stress, suggesting that target modules are more robust against random perturbation of regulatory networks.

## Methods

### Regulatory network data sets

A regulatory network involved in secondary cell wall synthesis [4] and a SHORTROOT-SCARECROW regulatory network [5] for *A. thaliana* were downloaded from online supplementary materials. These two regulatory networks were merged and duplicated interactions were removed. To test CoReg in a larger network of *Arabidopsis*, we downloaded a recent large-scale regulatory network generated by DAP-seq for *A. thaliana* [7]. The *E. coli* regulatory network data was downloaded from http://www.mrc-lmb.cam.ac.uk/genomes/madanm/ec_tf/ [3], which integrated TF-DNA interactions for *E. coli* from multiple publications. A *H. sapiens* network generated by ChIP-seq was downloaded from http://encodenets.gersteinlab.org/ [1]. For *H. sapiens*, we used only the interactions between the TFs and proximal promoters. Self-loops and duplicated edges were removed using the igraph R package [46]. However, self-loops and duplicated edges could be integrated into our computational tool with additional effort. In summary, there are 412 genes and 1490 edges in the *A. thaliana* yeast-1-hybrid network, 32,606 genes and 2,848,929 edges in the *A. thaliana* DAP-seq generated network, 889 genes and 1405 edges in the *E. coli* network, and 9057 genes and 26,043 edges in the *H. sapiens* network*.*

### CoReg method

#### Definition of directed networks

A regulatory network can be represented by a directed graph *G* = (*V*, *E*), where *V* (set of nodes) is the set of genes in the network and *E* (set of edges) is the set of TF-gene interactions. In a directed graph, each edge is represented by a pair of ordered nodes including a head node and a tail node. The head node is a TF (a regulator), whereas the tail node (a target) is the gene regulated by the head node and can be either a TF gene or a non-TF gene. Every edge *e* = (*i*, *j*), (*e* ∈ *E*) in *G* links a head node *v*_*i*_ to a tail node *v*_*j*_ (*v*_*i*_, *v*_*j*_ ∈ *V*). Here, the node *v*_*i*_ is an in-neighbor of *v*_*j*_, and *v*_*j*_ is an out-neighbor of *v*_*i*_.

#### Define problem

In a directed network with |*V*| nodes, a module *m* is a group of *n* nodes that is represented by *m* = {*v*_1_, *v*_2_, …*v*_*n*_| *v* ∈ *V*, *n* ≤ |*V*|}. When there are *M* modules in the graph, a partition *P* of the graph is a set of modules that divides all the nodes *V* into *M* modules. The goal of CoReg, is to find a partition *P* such that in each module *m*, for any two genes *v*_*i*_ and *v*_*j*_ (*v*_*i*_, *v*_*j*_∈ *m*), the similarity *S*(*v*_*i*_, *v*_*j*_) is greater than *S*(*v*_*i*_, *v*_*k*_) and *S*(*v*_*j*_, *v*_*k*_), (*v*_*k*_ ∉ *m*). Here, we tested several similarity scores and clustering approaches to find this partition. We also validated our methods by analyzing gene co-expression data.

CoReg takes a regulatory network (a directed graph) as input and generates a module assignment for each gene. CoReg first calculates a pairwise similarity score for all the nodes in *G* to generate a similarity score matrix ***S***. Then ***S*** is transformed into a dissimilarity matrix ***S***′. CoReg applies hierarchical clustering followed by the dynamic tree cut algorithm [47] to identify the modules. Figure 1b shows the brief workflow for CoReg. The detailed description for each step is given below. We evaluated CoReg using NMI, ROC curve, and rewiring recall score (See Performance Assessment in Methods section).

#### Similarity indices

We explored different similarity indices for calculating the pairwise similarity score. Three similarity indices were compared in this study: the Jaccard similarity index [48], the geometric similarity index [48], and the inverse log-weighted similarity index [39]. Given node *v*_*i*_, we define the set of in-neighbors and out-neighbors of *v*_*i*_ as ***N*** ^(*in*)^(*v*_*i*_) and ***N*** ^(*out*)^(*v*_*i*_). For every node pair *v*_*i*_ and *v*_*j*_, we computed in-similarity and out-similarity separately. For each pair of nodes, the **Jaccard index** is calculated by dividing the number of common neighbors by the total number of neighbors for both nodes:

$$ {J}_{v_i,{v}_j}^{(in)}=\frac{\mid {N}^{(in)}\left({v}_i\right)\cap {N}^{(in)}\left({v}_j\right)\mid }{\mid {N}^{(in)}\left({v}_i\right)\cup {N}^{(in)}\left({v}_j\right)\mid } $$

$$ {J}_{v_i,{v}_j}^{(out)}=\frac{\mid {N}^{(out)}\left({v}_i\right)\cap {N}^{(out)}\left({v}_j\right)\mid }{\mid {N}^{(out)}\left({v}_i\right)\cup {N}^{(out)}\left({v}_j\right)\mid } $$

The **geometric index** is the square of the number of common neighbors of *v*_*i*_ and *v*_*j*_ divided by the product of the number of neighbors of *v*_*i*_ and *v*_*j*_:

$$ {G}_{v_i,{v}_j}^{(in)}=\frac{{\left|{N}^{(in)}\left({v}_i\right)\cap {N}^{(in)}\left({v}_j\right)\right|}^2}{\left|{N}^{(in)}\left({v}_i\right)\right|\bullet \mid {N}^{(in)}\left({v}_j\right)\mid } $$

$$ {G}_{v_i,{v}_j}^{(out)}=\frac{{\left|{N}^{(out)}\left({v}_i\right)\cap {N}^{(out)}\left({v}_j\right)\right|}^2}{\left|{N}^{(out)}\left({v}_i\right)\right|\bullet \mid {N}^{(out)}\left({v}_j\right)\mid } $$

For the Jaccard similarity index, if both *v*_*i*_ and *v*_*j*_ have no common in-neighbors/out-neighbors, the corresponding in-similarity/out-similarity score is set to zero. For the geometric similarity index, if either *v*_*i*_ or *v*_*j*_ has no in-neighbors/out-neighbors, the corresponding in-similarity/out-similarity score is set to zero.

The **inverse log weighted similarity index** is the inverse log weighted sum of the degree of all the common neighbors for *v*_*i*_ and *v*_*j*_. The idea is that two nodes may be more similar if they share some common low-degree neighbors, because high-degree neighbors are more likely to connect to the nodes by pure chance [39]. The degree of the node c is represented by *d(c)*.

$$ {I}_{v_i,{v}_j}^{\left(\mathrm{in}\right)}=\sum \limits_{c\in \left\{{N}^{(in)}\left({v}_i\right)\cap {N}^{(in)}\left({v}_j\right)\right\}}\frac{1}{\mathit{\log}\left(d(c)\right)} $$

$$ {I}_{v_i,{v}_j}^{\left(\mathrm{out}\right)}=\sum \limits_{c\in \left\{{N}^{(out)}\left({v}_i\right)\cap {N}^{(out)}\left({v}_j\right)\right\}}\frac{1}{\mathit{\log}\left(d(c)\right)} $$

For weighted networks, the degree of each node can be replaced by the sum of edge weights.

#### Similarity and dissimilarity matrix

CoReg calculates the similarity score between every pair of *v*_*i*_ and *v*_*j*_ to get in- and out-similarity matrices, respectively. Similarity matrix ***S*** is the sum of two matrices. A dissimilarity matrix ***S′*** was computed based on ***S***:

$$ {\boldsymbol{S}}_{ij}={\boldsymbol{w}}_1{\boldsymbol{S}}_{ij}^{\left(\mathrm{in}\right)}+{\boldsymbol{w}}_2{\boldsymbol{S}}_{ij}^{\left(\mathrm{out}\right)} $$

$$ {\boldsymbol{S}}_{ij}^{\prime }=\frac{\mathit{\max}\left(\boldsymbol{S}\right)-{\boldsymbol{S}}_{ij}}{\mathit{\max}\left(\boldsymbol{S}\right)} $$

where ${\boldsymbol{S}}_{ij}^{\left(\mathrm{in}\right)}$ is the incoming similarity between gene *v*_*i*_ and *v*_*j*_ and ${\boldsymbol{S}}_{ij}^{\left(\mathrm{out}\right)}$ the outgoing similarity between *v*_*i*_ and *v*_*j*_. The maximum value in matrix ***S*** is denoted by max(***S***). The values ***w***_1_ and ***w***_2_ represent weights assigned to the incoming and outgoing similarity matrices. In this study, we set ***w***_1_ = ***w***_2_ =1.

#### Hierarchical clustering and dynamic tree cut

Hierarchical clustering and dynamic tree cut is implemented using the R built-in function "hclust" and the R package DynamicTreeCut [47]. Hierarchical clustering was performed on the dissimilarity matrix with complete linkage as the agglomeration method. The R package DynamicTreeCut [47] was then used to cut the tree from hierarchical clustering with a "hybrid" cutting method. The advantage of the dynamic tree cut algorithm over the fixed height tree cut is that the dynamic tree cut method takes into account the shape of the hierarchical tree and cuts the tree adaptively. In brief, the core of a cluster consists of nodes that have low dissimilarity with each other in the cluster. The core scatter is the average of pairwise dissimilarity in the core, and the cluster gap is the difference between core scatter and the joining height where the cluster joins the dendrogram. The dynamic tree cut algorithm merges the clusters in the dendrogram from bottom to top. When the criterion of core scatter and cluster gap is met, the cluster will stop merging with other clusters. This process will continue until all the clusters stop merging. We chose dynamic tree cut to process the dendrogram, because, when no prior knowledge except for the network itself is provided, it is difficult to find a single unique cutting height that works best. Here, we set the minimum size of a cluster to 2, since the co-regulation activity requires at least two genes to collaborate. The R package DynamicTreeCut provides a parameter "deepSplit" to conveniently set up the threshold for cluster shape. The deepSplit parameter takes only four values, 0, 1, 2, 3. The higher the value is, the smaller the clusters tend to be. For the analysis in this paper, we used the default value for deepSplit (deepSplit = 1). However, other values for deepSplit can be set in our CoReg package. Please refer to the supplementary materials of [47] for more details about the algorithm.

#### Performance assessment

##### Overview of performance assessment

In the following subsections, we described the bipartite transformation, generating simulated co-regulatory network, rewiring simulation, estimation of AUC and ROC, and comparison between different tree cutting methods. Bipartite transformation was performed to transform a directed network to an undirected network so that LP, EB, and WT can be performed on this transformed network. Simulated co-regulatory networks were used to assess the ability of different methods to identify pre-specified co-regulatory modules. Rewiring simulation and estimation of AUC and ROC were designed to assess the performance of different module finding methods. Performance of different tree cutting methods was compared to find the best strategy for co-regulatory module finding. The network is first randomly rewired and different module finding methods were applied to the rewired network to generate modules. A performance score is computed based on the module finding result. AUC and ROC were estimated using the similarity score calculated from the rewired network. Figure 1d illustrates the flow chart of the analyses.

##### Bipartite transformation for LP, EB, and WT

To compare the performance of CoReg to other module finding algorithms, we applied CoReg, LP, EB, and WT algorithms on the same data. However, LP, EB, and WT were initially designed for undirected networks and cannot be directly applied to directed networks. This could be solved by transforming the directed network into a bipartite network [15]. Such an undirected network preserves the direction information [15]. In this paper, we applied a transformation process as described previously [15]. A bipartite network is defined as *G*_*B*_ = (*V*_*h*_, *V*_*t*_, *E*_*b*_) and *G*_*B*_ is transformed from a directed network *G* = (*V*, *E*) according to:

$$ {V}_h=\left\{{v}_h|v\in V,{k}_v^{out}>0\right\} $$

$$ {V}_t=\left\{{v}_t|v\in V,{k}_v^{in}>0\right\} $$

where *V*_*h*_ is the set of nodes transformed from source nodes in *G* and *V*_*t*_ is the set of nodes transformed from target nodes in *G* and *E*_*b*_ is the set of edges in *G*_*B*_. ${k}_v^{in}$ and ${k}_v^{out}$ are the in-degree and out-degree for node *v*_*h*_ and node *v*_*t*_, respectively. For each directed edge *u* → *v* in *G*, we transformed *u* into a head node *u*_*h*_ and transformed *v* into tail node *v*_*t*_. Then *u*_*h*_ and *v*_*t*_ will be added into *V*_*h*_ and *V*_*t*_, respectively. Then the undirected edge between *u*_*h*_ and *v*_*t*_ is created. Additional file 9: Figure S4 shows an example of transforming the directed network into a bipartite network. Next, we applied the LP, EB, and WT algorithms on the bipartite networks, assigning the module to *V*_*h*_ and *V*_*t*_. If the head node and the tail node were transformed from the same node but were given different modules, we assigned to this node all the modules that were assigned to both the head node and the tail node. We implemented the bipartite transformation and LP, EB, and WT using the igraph R package [46].

##### Generating simulated co-regulatory network

To assess the performance of different module-finding methods, we generate ground-truth modules by constructing a simulated network. The process is similar to a published method [21]. The original method was proposed to generate modules for a bipartite network. Here, we modified this approach to generate co-regulatory modules in a directed network. The simulation requires five parameters:

1. *mSize*: size of each module
2. *mNum*: total number of modules
3. *targetNum*: number of targets for each regulator node
4. *auxNum*: number of auxiliary nodes
5. *prob*: co-regulation probability.

We constructed simulated networks by the following steps:

1. Generate *mSize × mNum* regulator nodes. Each node is assigned to one module and each module will have an equal number of nodes (specified by *mSize*).
2. Generate auxiliary nodes as specified by *auxNum*. These nodes are targets of regulators and will not have any outgoing edges.
3. Select a pool of target candidates for each module. The size of pool is equal to *targetNum*. Each pool is considered as a set of potential co-regulated targets for the corresponding module.
4. For each regulator node, with probability *prob*, randomly select a target from the pool of target genes. Otherwise, randomly select a target not in the pool. With higher *prob*, regulator nodes in the same module will tend to select nodes from the pool of target genes, showing stronger co-regulating modular structure (Additional file 1: Figure S1). The numbers of targets for regulators are the same (specified by *targetNum*). These steps will be repeated until all regulator nodes have been assigned the given number of targets.

The simulated network is generated as an edge list. The pseudocode implementation is provided in Additional file 10: Table S6. In our simulation experiment, we explored the different settings of parameters. We set *mSize* = (2, 5, 15, 20), *mNum* = 10, *targetNum* = (5, 15, 20), *auxNum* = 200, *prob* = (0, 0.2, 0.4, 0.6, 0.8, 1). We set *mSize* = (2, 5, 15, 20), because the number of known transcription co-regulators are usually small. We set *targetNum* = (5, 15, 20), *mNum* = 10, and *auxNum* = 200, such that the total number of genes in the network is similar to the number observed in the *Arabidopsis* Y1H network. We set *targetNum, mNum,* and *auxNum* also, because we want to reduce the total time of computation. Higher *targetNum* or *auxNum* are likely to lead to better performance, because the calculation of similarity will be more stable with more nodes. Higher *mNum* is unlikely to alter the performance. We used the NMI score to quantify the correlation between pre-specified modules and algorithm identified modules [22].

##### Network duplication and rewiring

As no published genome scale studies of true co-regulators are available, we constructed the true co-regulators by duplicating nodes in the network. We also introduce noise to the network by rewiring simulation. For a given network ***G***, we randomly selected **n** nodes from the network and duplicated these nodes. If we duplicate a small number of nodes, there will not be sufficient nodes for performance analysis. If we duplicate too many nodes, the duplicated network will drastically alter the topology of the original network. Because of these restrictions, we chose to set *n* = 80 nodes in each of the networks. We define the set of randomly selected original nodes as ***U*** and the set of their duplicated nodes as ***U*** ^′^. The corresponding nodes in ***U*** and ***U*** ^′^ were denoted as *u* and *u* ^′^. For each duplicated node *u*_*i*_^′^, the neighbors of *u*_*i*_ were set as neighbors for *u*_*i*_^′^. For these duplicated nodes, each node and its original node are a pair of “true” co-regulators (i.e. *u*_*i*_ -> *u*_*i*_^′^). Negative co-regulators were defined by randomly selecting one from the duplicated nodes and another from duplicated nodes of other nodes.

We rewired the edges connecting *u*_*i*_^′^ to its neighbors with a selected probability. One edge starting from *u*_*i*_^′^ will be randomly selected with given probability, then another edge starting from another duplicated node in ***U*** ^′^ will be randomly selected as well. The target nodes of the two edges will be swapped. Additional file 11: Figure S5 shows the steps of rewiring in detail. The rewiring operations do not change the total number of interactions or the node degree distributions in the network. In our rewiring simulation, we also preserved the topology of the original networks by rewiring only duplicated nodes. We did not perform rewiring simulation in the whole network, because if we rewire the whole network with a given probability, the chance of rewiring the newly duplicated nodes is very low.

##### Calculate rewiring recall score

To compare the module finding result of CoReg to WT, LP, and EB, we calculated rewiring recall scores according to following equations:

7 $$ score=\frac{\sum^{u_i\in \boldsymbol{U}}{s}_{u_i}{w}_{u_i}}{\frac{\mid \boldsymbol{V}\mid \mid \boldsymbol{U}\mid }{2}} $$

8 $$ {w}_{u_i}=\frac{\mid \boldsymbol{V}\mid }{m_{u_i}} $$

where ${s}_{u_i}$ = 1 if *u*_*i*_ and *u*_*i*_^′^ are in the same module otherwise ${s}_{u_i}$ = 0. ∣***U***∣ is total number of nodes in ***U***. ∣***V***∣ is total number of nodes in the network. Here, ${m}_{u_i}$ is the number of nodes in the module of *u*_*i*_ . Here, ${w}_{u_i}$ is a weight to reduce the effect of false positives due to module size, since the larger the module size is, the more probable that the module will include both *u*_*i*_ and *u*_*i*_^′^ by pure chance. The denominator of eq. (7) is the theoretical maximum value of the numerator. The rewiring recall score is a number between zero and one, and the score will be equal to one when every pair of *u*_*i*_ and *u*_*i*_^′^ is in the same module and the module contains only *u*_*i*_ and *u*_*i*_^′^.

##### Similarity between co-regulators for walk trap method

For Walk Trap, we calculated the similarity by two steps. First, we converted the regulatory network to a directed adjacency matrix ***A***, which preserved direction information. If node *v*_*i*_ points to node *v*_*j*_, we mark the entry ***A***_*ij*_ as ‘1’, otherwise ‘0’. All the diagonal entries were set to ‘1’ to add self-loops to avoid being divided by 0 in the walk trap algorithm. Second, we calculated a transition matrix *T* according to the equation below:

$$ {\boldsymbol{T}}_{ij}=\frac{{\boldsymbol{A}}_{ij}}{\sum_{k=1}^{\mid \boldsymbol{V}\mid }{\boldsymbol{A}}_{ik}} $$

Where the denominator is the out degree of node *v*_*i*_. We calculated a probability matrix similar to what was described in [17]. Here, the probability matrix is ***P*** = ***T*** ^*m*^, and we set m = 2 and 4. Similar to [17], we calculated the distance between the co-regulators *v*_*i*_ and *v*_*j*_ using the following equation:

$$ D\left({v}_i,{v}_j\right)=\sqrt{\sum_{k=1}^{\mid \boldsymbol{V}\mid}\frac{{\left({\boldsymbol{P}}_{ik}-{\boldsymbol{P}}_{jk}\right)}^2}{d\left({v}_i\right)}} $$

where *d*(*v*_*i*_) denotes the degree of node i and n is the total number of nodes. Here, *D*(*v*_*i*_, *v*_*j*_) defines the pairwise distance between node *v*_*i*_ and *v*_*j*_. Similarity for Walk Trap is therefore defined as ***S***_*ij*_ = 1 − *D*(*v*_*i*_, *v*_*j*_). We ranked the co-regulators pairs using the above-mentioned similarity measurement then computed ROC curves and AUC values.

#### Comparison between dynamic tree cut and static tree cut

We first performed the duplication and rewiring process as described in Methods section. Then we calculated a distance matrix and applied hierarchical clustering. In the next step, we applied both a static tree cut method and a dynamic tree cut method. For the static tree cut, we sampled the cutting height from 0 to 1 (1 is the maximum tree height since distance ranges from 0 to 1) and increased the cutting height by 0.1 each time (11 sampled points in total). We evaluated each cutting height using RRS and then picked the highest RRS for each rewiring probability. For the dynamic tree cut, two parameters were used to find the optimal RRS: maximum cutting height and deepSplit. The sampling process for maximum cutting height is the same as the sampling process for cutting height in static tree cut. The deepSplit parameter only has five possible values (0,1,2,3,4). Therefore, a grid of 11 × 4 combinations was searched to find the optimal RRS. K-means clustering was added to compare to these two strategies. The parameter k ranges from 1 to the number of nodes in the network and increased by (number of nodes)/10 each time. This produced 11 RRS for each rewiring probability. The highest RRS was then picked as the optimal score.

#### Co-expression analysis

##### Expression data sets

We downloaded expression data sets for *A. thaliana* under stress [25], hormone [24], and developmental condition [26]. The stress expression data set was generated using *A. thaliana* exposed to various abiotic stresses including heat, cold, drought, salt, high osmolarity UV-B light, and wounding [25]. The hormone expression data set was produced from *A. thaliana* samples treated with auxin, cytokinin, gibberellin, brassinosteroid, abscisic acid, jasmonate,the and ethylene [24]. Gene expression in developmental data set was detected from *A. thaliana* in a series of developmental stages [26]. To compute the co-expression level of coReg-identified modules in genome-scale network, we downloaded over 2300 gene expression samples from a recent publication that collected over 6000 expression samples in total for *A. thaliana* [45]. We selected the experiments that contain more than 10 conditions for co-expression analysis.

##### Co-expression and *p*-value calculation

We used expression data to estimate the correlations between co-regulators. For each pair of co-regulators, we calculated the Pearson Correlation Coefficient (PCC) of expression. To estimate the module-level significance of correlation, for each co-regulator module, we calculated the average PCC over all the co-regulators pairs and randomly selected the same number of genes from the whole genome and calculated average PCC. This step was repeated 1000 times. Thus, the *p*-value of PCC is defined as how many times the random PCC is higher than the actual PCC of a given module.

##### Estimate *p*-value in large-scale network

Since the number of expression data sets and the number of modules are large for the DAP-seq network, it is computationally expensive to compute a *p*-value by random permutation as described previously. Here, we computed the *p*-value using Fisher’s combined probability test. Briefly, we first randomly selected two genes from the genome and calculated their co-expression value. This was repeated for 10,000 times to generate an empirical null distribution of pairwise co-expression. Then for each module, we calculated the pairwise *p*-value for all the genes in the module. A module level statistic combining these *p*-values is calculated using the following equation:

$$ {X}_{2k}^2=-2\sum \limits_{i=1}^k\ln \left({p}_i\right) $$

Where *k* is the total number of *p*-values and *p*_*i*_ is the *i*th p-value for the module. The statistic ${\mathrm{X}}_{2k}^2$ follows the χ^2^ distribution with 2 k degrees of freedom. The module level *p*-value was then computed using the statistic ${\mathrm{X}}_{2k}^2$ and the χ^2^ distribution.

## Additional files

Additional file 1: Figure S1. Co-regulation pattern in networks with different co-regulation probability (specified by the parameter *prob*). Network with higher *prob* is expected to have stronger co-regulation pattern. We generated two modules ‘a’ and ‘b’ in this example network. Modules are marked by different color. Zero-degree auxiliary nodes were not shown in the Fig. A) network generated with *prob* = 0.1 B) network generated with *prob* = 0.9 (PDF 38 kb)

Additional file 2: Figure S2. Evaluation of different module-finding methods using simulated networks with different parameters. From top row to bottom row: mSize = 5, mSize = 15, mSize = 20. From left most column to right most column: targetNum = 5, targetNum = 15, targetNum = 20. (PDF 73 kb)

Additional file 3: Figure S3. Rewiring recall score for LP, WT and EB in real networks. We rescaled the y-axis to highlight the differences in the curves for LP, WT and EB (PDF 29 kb)

Additional file 4: Table S1. Module identified by CoReg in yeast-1-hybrid network. 0 means no module assignment (XLSX 18 kb)

Additional file 5: Table S2. Edges connecting each module member with their first neighbors (XLSX 193 kb)

Additional file 6: Table S3. Experiments used in co-expression analysis of DAP-seq network (XLSX 12 kb)

Additional file 7: Table S4. Co-expression levels (PCC) for each CoReg modules. This table is ordered in the same way as the heatmap (XLSX 92 kb)

Additional file 8: Table S5. *P-*values for co-expression analysis for Fig. 7 heatmap. (XLSX 96 kb)

Additional file 9: Figure S4. The example of bipartite transformation. Network on the left is a directed network, which could be transformed into a bipartite network on the right. The suffix ‘_h’ represents the head node and ‘_t’ means the tail node (PDF 11 kb)

Additional file 10: Table S6. Algorithm: Generate simulated network (PDF 355 kb)

Additional file 11: Figure S5. Network duplication and rewiring. We randomly selected a subset of nodes from the whole network then duplicated them. New nodes that were duplicated from the original nodes are referred as ‘pseudo node’. In the figure, A^′^ and E^′^ are the pseudo nodes of A and E, respectively. This means before rewiring occurs, A^′^ and E^′^ duplicated all the edges from A and E (These duplicated edges are the dashed edges in the figure). For rewiring, CoReg first goes through every edge connecting to A^′^ and attempts to rewire the edge with given probability. Once CoReg decides to rewire that edge, another edge in the network will be randomly selected. Then the target nodes for these two edges will be exchanged. In the case shown above, the two edges marked by red box have their target nodes swapped. Therefore, rewiring only applies on pseudo nodes and the original graph remains unchanged during the process (PDF 27 kb)
