# Supplementary material for: Identification of regulatory modules in genome scale transcription regulatory networks
Source: BMC Syst Biol. 2017 Dec 15;11:140. doi: 10.1186/s12918-017-0493-2 (PMC5732458; doi:10.1186/s12918-017-0493-2)
Supplement: Supplementary file 1 — Co-regulation pattern in networks with different co-regulation probability (specified by the parameter prob). Network with higher prob is expected to have stronger co-regulation pattern. We generated two modules ‘a’ and ‘b’ in this example network. Modules are marked by different color. Zero-degree auxiliary nodes were not shown in the Fig. A) network generated with prob = 0.1 B) network generated with prob = 0.9 (PDF 38 kb) [file 12918_2017_493_MOESM1_ESM.pdf]

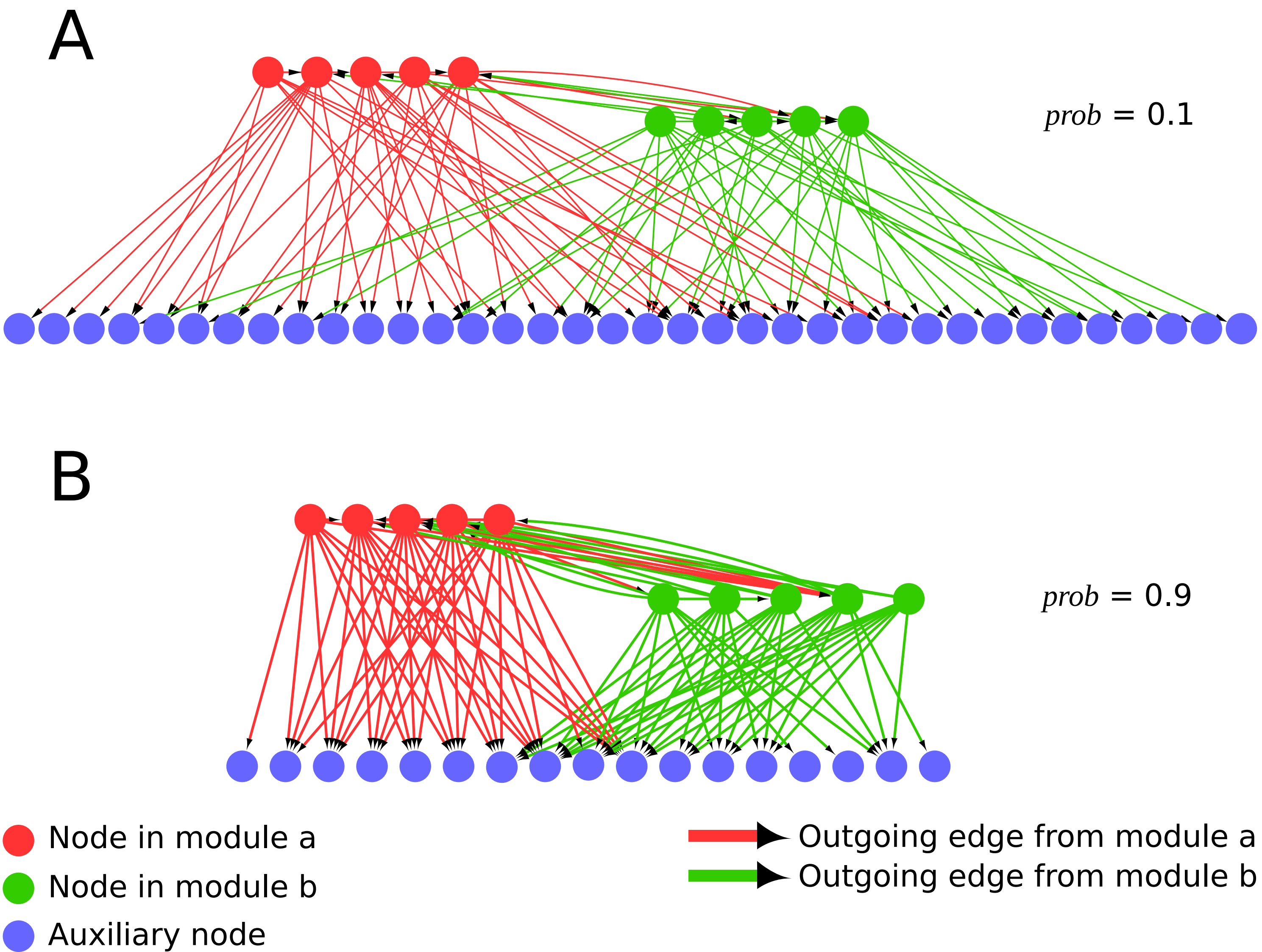
A
prob = 0.1
B
prob = 0.9
Node in module a
Node in module b
Auxiliary node
Outgoing edge from module a
Outgoing edge from module b
