# Supplementary figures and images for: Identification of regulatory modules in genome scale transcription regulatory networks
Source: BMC Syst Biol. 2017 Dec 15;11:140. doi: 10.1186/s12918-017-0493-2 (PMC5732458; doi:10.1186/s12918-017-0493-2)

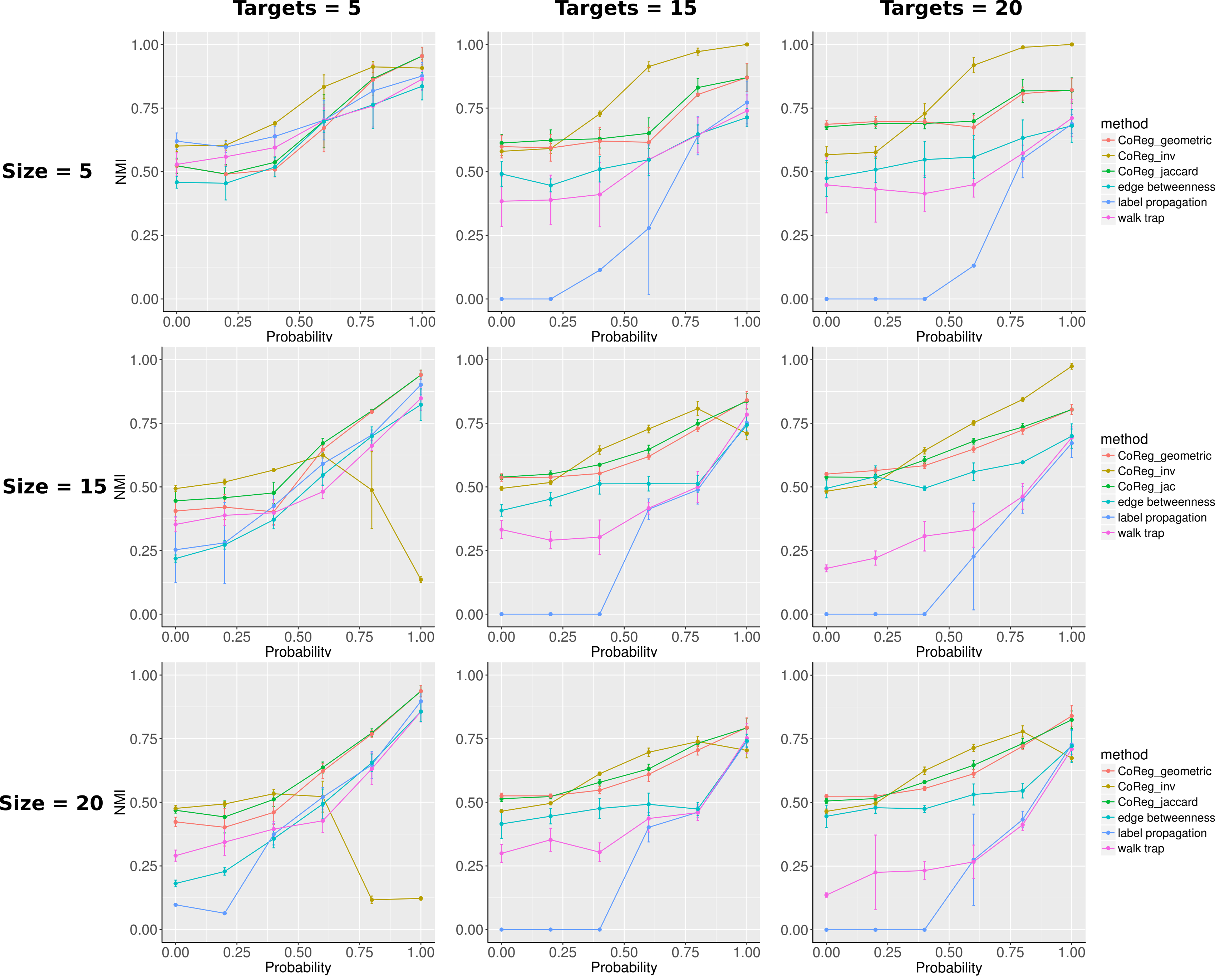

Supplement: Supplementary file 2 — Evaluation of different module-finding methods using simulated networks with different parameters. From top row to bottom row: mSize = 5, mSize = 15, mSize = 20. From left most column to right most column: targetNum = 5, targetNum = 15, targetNum = 20. (PDF 73 kb) [file 12918_2017_493_MOESM2_ESM.pdf]

**A** *A. thaliana*

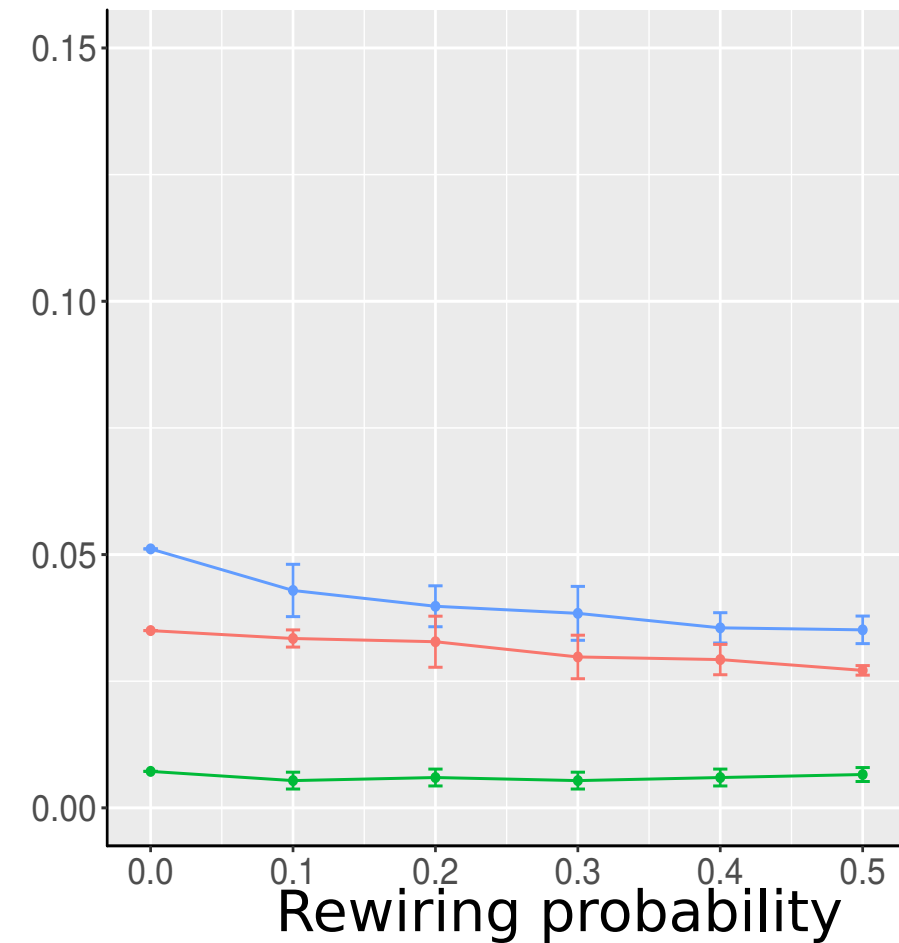

**B** *E. coli*

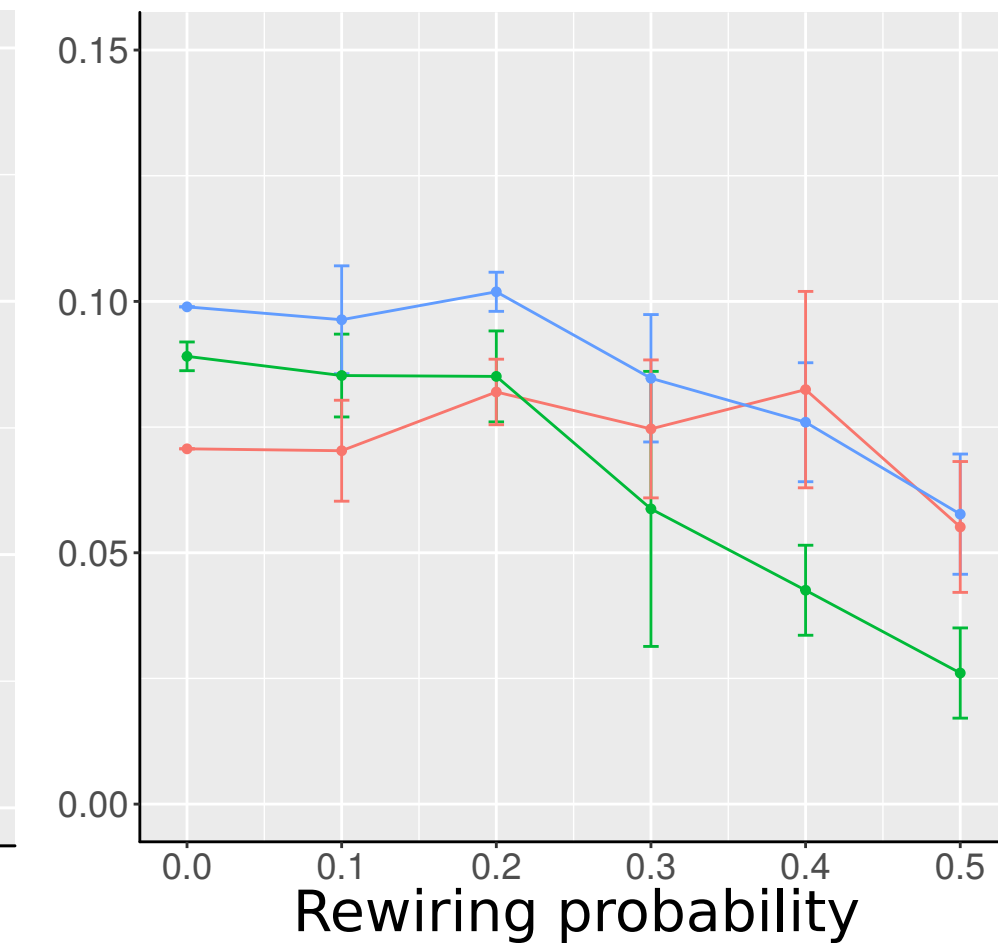

**C** *H. sapiens*

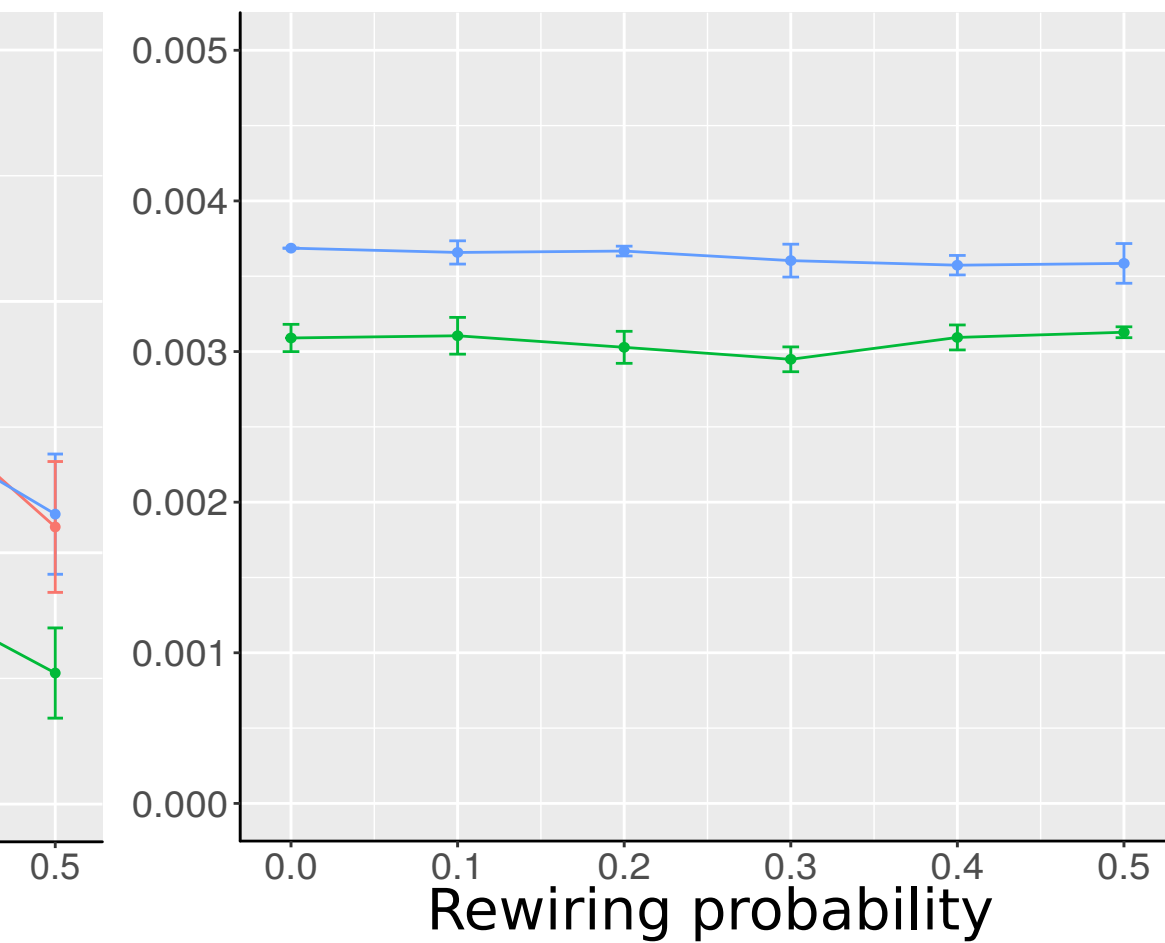

method

- edge betweenness
- label propagation
- walk trap

Supplement: Supplementary file 3 — Rewiring recall score for LP, WT and EB in real networks. We rescaled the y-axis to highlight the differences in the curves for LP, WT and EB (PDF 29 kb) [file 12918_2017_493_MOESM3_ESM.pdf]

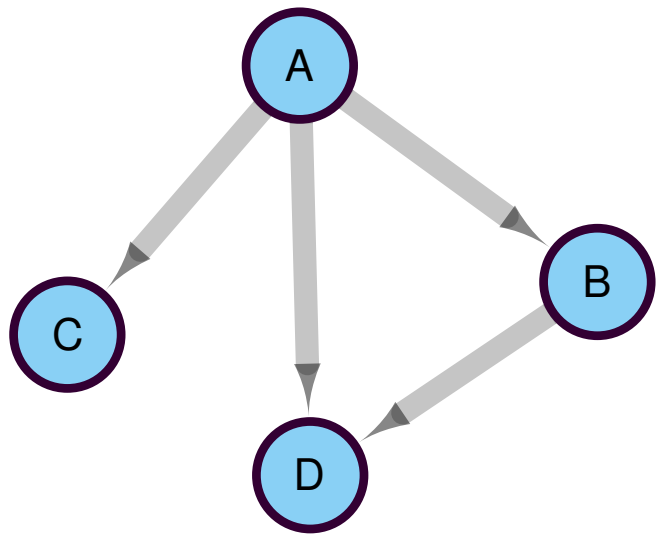

Directed network

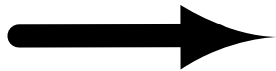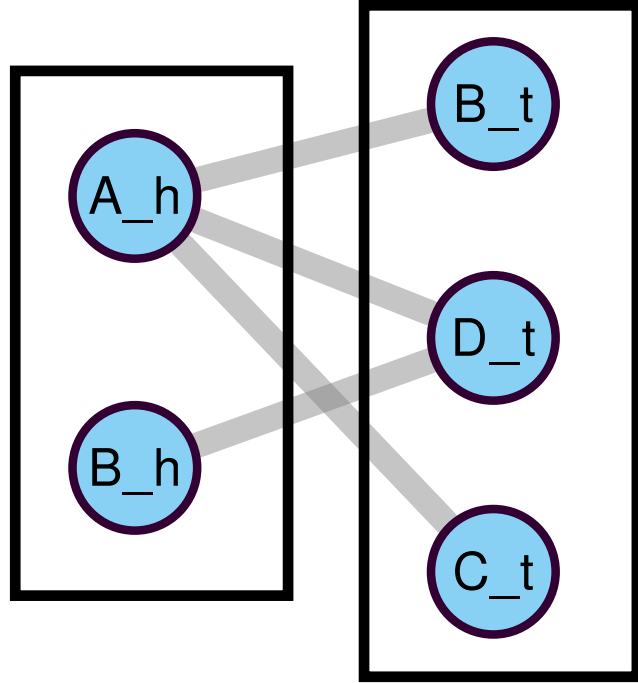

Bipartite network

Supplement: Supplementary file 9 — The example of bipartite transformation. Network on the left is a directed network, which could be transformed into a bipartite network on the right. The suffix ‘_h’ represents the head node and ‘_t’ means the tail node (PDF 11 kb) [file 12918_2017_493_MOESM9_ESM.pdf]

1. original graph

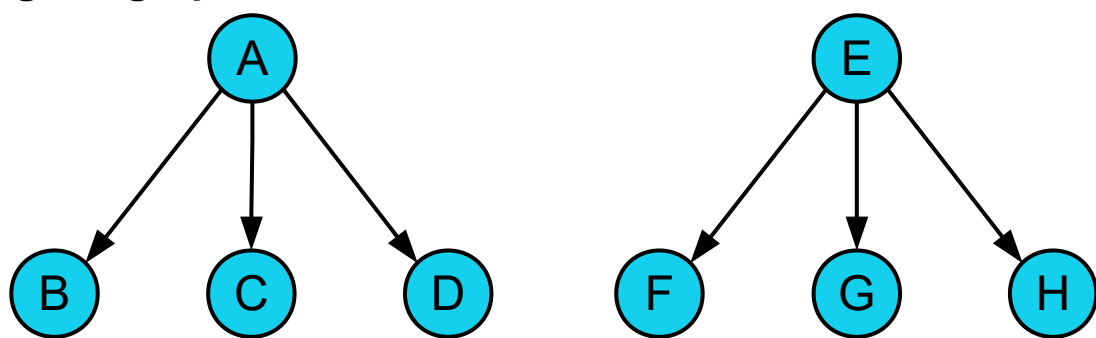

2. duplication

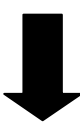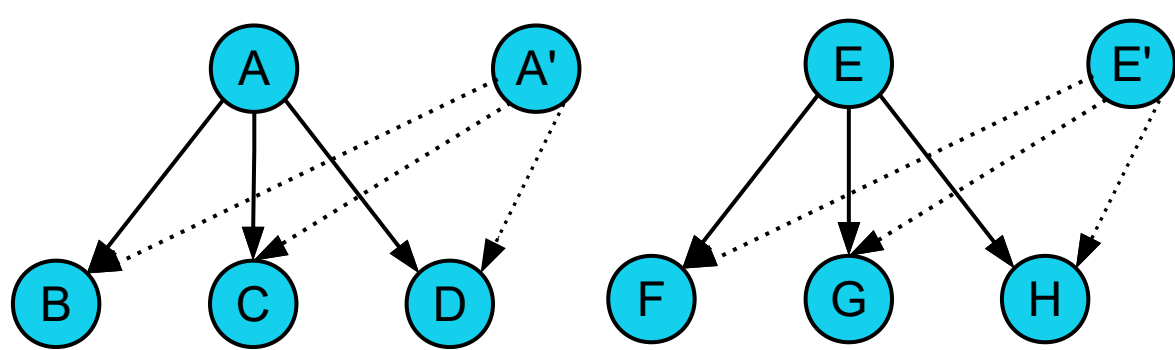

3. randomly select edges

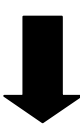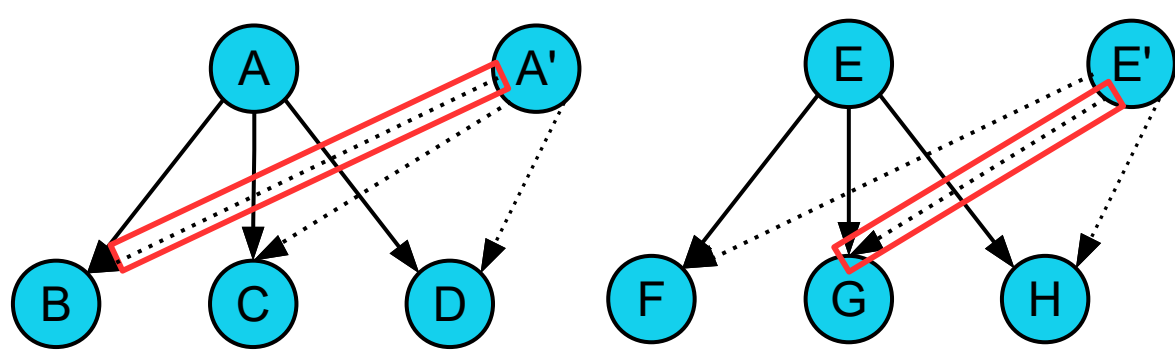

4. swap targets

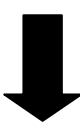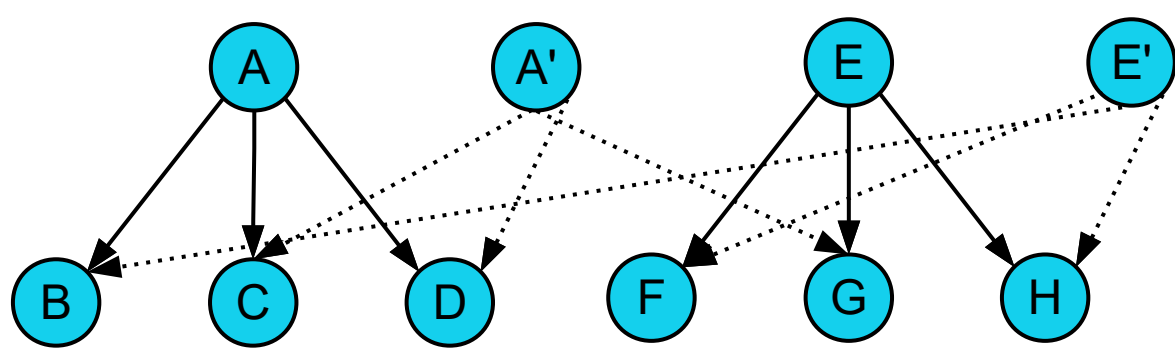

Supplement: Supplementary file 11 — Network duplication and rewiring. We randomly selected a subset of nodes from the whole network then duplicated them. New nodes that were duplicated from the original nodes are referred as ‘pseudo node’. In the figure, A′ and E′ are the pseudo nodes of A and E, respectively. This means before rewiring occurs, A′ and E′ duplicated all the edges from A and E (These duplicated edges are the dashed edges in the figure). For rewiring, CoReg first goes through every edge connecting to A′ and attempts to rewire the edge with given probability. Once CoReg decides to rewire that edge, another edge in the network will be randomly selected. Then the target nodes for these two edges will be exchanged. In the case shown above, the two edges marked by red box have their target nodes swapped. Therefore, rewiring only applies on pseudo nodes and the original graph remains unchanged during the process (PDF 27 kb) [file 12918_2017_493_MOESM11_ESM.pdf]
