# Supplementary material for: Identification of regulatory modules in genome scale transcription regulatory networks
Source: BMC Syst Biol. 2017 Dec 15;11:140. doi: 10.1186/s12918-017-0493-2 (PMC5732458; doi:10.1186/s12918-017-0493-2)
Supplement: Supplementary file 10 — Algorithm: Generate simulated network (PDF 355 kb) [file 12918_2017_493_MOESM10_ESM.pdf]

Algorithm: Generate simulated network

Input: *mSize*: module size
mNum: total number of modules to be generated
targetNum: number of targets for each node in module
auxNum: number of auxiliary nodes
prob: probability of selecting a target from target candidate pool
Output: An edge list *el* and an array *partition* for ground-truth module partition

```
genSimNet(mSize, mNum, targetNum, auxNum, prob)
    // Initialization
    partition ← empty array
    el ← empty matrix
    nodeID ← 1 to (mSize*mNum+auxNum)

    // Initialize ground-truth module partition
    for i = 1 to mNum
        for j = 1 to mSize
            partition.append(i)

    // Select targets for each node whose ground-truth module is prespecified
    currentID ← 1
    for i = 1 to mNum
        pool ← randomly sample targetNum targets from nodeID    // Target candidate pool
        for j = 1 to mSize
            for k = 1 to targetNum
                p ← rand()
                if p <= prob
                    targetID ← randomly sample a target from pool
                else
                    targetID ← randomly sample a target from nodes not in pool
                el.appendRow(currentID, targetID)
            currentID ← currentID + 1
    return el, partition
```
